# Wildland fire smoke and birth defects in California

**DOI:** 10.1038/s41370-026-00885-4

**Published:** 2026-04-15

**Authors:** Amy M. Padula, Jonathan A. Mayo, Fred W. Lurmann, Nathan R. Pavlovic, Gary M. Shaw

**Affiliations:** 1 Department of Obstetrics, Gynecology and Reproductive Sciences, University of California, San Francisco, San Francisco, CA USA; 2 Department of Pediatrics, Stanford University School of Medicine, Palo Alto, CA USA; 3 Sonoma Technology Inc., Petaluma, CA USA

**Keywords:** Air pollution, Children’s health, Early life exposure, Epidemiology, Health studies, Maternal and fetal exposure/health, Wildfires

## Abstract

**Background:**

Birth defects are a leading cause of infant mortality and an important contributor to childhood and adult morbidity. Major structural birth defects are diagnosed in 2 to 4% of infants in the United States. Environmental contaminants, including air pollution, have been suggested as potential risk factors for these anomalies.

**Objective:**

This study investigates the association between wildfire smoke exposure, specifically fine particulate matter (PM_2.5_), and the risk of selected structural birth defects in the San Joaquin Valley of California.

**Methods:**

Data were collected from participants in the California Center of the National Birth Defects Prevention Study and the Birth Defects Study to Evaluate Pregnancy exposureS from 2000 to 2018. The study included 3005 participants, comprising livebirths, stillbirths, and pregnancy terminations. Wildfire smoke exposure was assessed using two estimates: fire density PM_2.5_ and fire increment PM_2.5_. Logistic regression was used to analyze the odds of 14 birth defects in relation to quartile-based exposure metrics during the first 8 weeks of pregnancy, adjusting for relevant covariates.

**Results:**

Increased risk for several birth defects was observed with higher quartiles of wildfire smoke exposure. Notably, the third quartile of average fire density PM_2.5_ was associated with increased odds of cleft lip (OR = 2.29; 95% CI: 1.12, 4.69), and the highest quartile of maximum fire density PM_2.5_ was associated with increased risk of coarctation of the aorta (OR = 2.26; 95% CI: 1.02, 4.99). Spina bifida was consistently associated with higher exposure across multiple metrics, including a significant association with the highest quartile of maximum fire increment PM_2.5_ (OR = 2.76; 95% CI: 1.14, 6.66).

**SIGNIFICANCE:**

Exposure to wildfire smoke during the first 8 weeks of pregnancy may contribute to the occurrence of certain structural birth defects in California’s San Joaquin Valley. These results highlight the need for further research to corroborate these findings and to understand the mechanisms underlying these associations.

**Impact statement:**

- This study examines the association between wildfire smoke exposure in California and risk of several structural birth defects. We found associations between wildfire smoke exposure during the first 8 weeks of pregnancy and increased risk of cleft lip, coarctation of the aorta and spina bifida. The association between wildfire smoke and spina bifida is consistent with prior research findings similar to those of air pollution from other sources, primarily traffic, in California.

## Introduction

Birth defects are a leading cause of infant mortality and an important contributor to childhood and adult morbidity. Major structural birth defects are diagnosed in 2 to 4% of infants [1]. Although some can be attributed to chromosomal abnormalities or known teratogenic agents, the etiologies of most cases remain unknown. Environmental contaminants have been suggested as risk factors for some anomaly groups; however, few studies have investigated structural birth defects in specific phenotypic groupings [2, 3].

In previous analyses we examined associations between traffic-related air pollution and neural tube defects, orofacial clefts, gastroschisis [4], several congenital heart defects [5], and other selected defects [6] with data from the California Center of the National Birth Defects Prevention Study [7] and found increased risks of spina bifida for those pregnancies exposed to higher levels of carbon monoxide and nitrogen dioxide. Wildfires are a significant and increasing source of air pollution in California (CA) and have reversed much of the air pollution reduction that began with the introduction of the Clean Air Act in the 1990s. Recent fires are occurring at the wildland-urban interface, the area between unoccupied land and human development, leading to increased biomass and structure burning. Particulate matter (PM) is possibly the most important health-related component of wildfire events [8]. Wildfire-specific PM may be more toxic, on an equal-mass basis, than ambient PM during non-fire periods [9]. In CA, half of the emissions of annual ambient fine PM, less than 2.5 microns in aerodynamic diameter (PM_2.5_), are attributed to wildfires, and this proportion is expected to increase in the coming decades [10–13]. This problem is not unique to CA—both wildfire size and intensity have quadrupled in the last four decades nationwide [14]. Furthermore, wildfire smoke can disperse thousands of miles from the source and cross geographical boundaries without active fires [15], and the San Joaquin Valley of California tends to trap air pollution between its surrounding mountain ranges and meteorological characteristics such as inversions [16, 17]. There are several existing methods to quantify wildfire smoke exposure; we will use two distinct methods to estimate exposure in our study population. The aim of the current analysis was to investigate whether air pollution attributable to wildland fire smoke, namely, fine particulate matter, contributed to the risks of selected structural anomalies in the San Joaquin Valley of California.

## Materials and methods

### Study population

Our study included participants from the California Center of the National Birth Defects Study (NBDPS) and Birth Defects Study To Evaluate Pregnancy exposureS (BD-STEPS) from 2000–2018. The Center collected data from women residing in 8 counties (San Joaquin, Stanislaus, Merced, Madera, Fresno, Kings, Tulare, and Kern) in the San Joaquin Valley through population-based surveillance by the California Birth Defects Monitoring Program. Highly trained data collection staff visit all hospitals to identify cases with birth defects. Staff members review and abstract cases, including those diagnosed prenatally with birth defects.

Eligible cases included livebirths (ascertained up to age 1), stillbirths, and pregnancy terminations and were selected from the center’s surveillance system based on strict eligibility criteria. Controls included nonmalformed liveborn infants randomly selected from birth hospitals to represent the population from which the cases arose. Maternal interviews were conducted by using a standardized, computer-based questionnaire, primarily by telephone, in English or Spanish, between 6 weeks and 24 months after the infant’s estimated date of delivery.

We included defects that had at least 40 cases in the combined NBDPS and BD-STEPS studies. Cases in the current analysis included infants with spina bifida, anotia/microtia, cleft palate, cleft lip with or without cleft palate, cleft lip alone (NBDPS data only), esophageal atresia, transverse limb deficiency, diaphragmatic hernia, gastroschisis, tetralogy of Fallot, D-Transposition of the great arteries, total anomalous pulmonary venous return (TAPVR), hypoplastic left heart syndrome, and coarctation of the aorta as confirmed by clinical, surgical, or autopsy reports. Cases resulting from known single gene or chromosomal abnormalities or with identifiable syndromes were ineligible, given their presumed distinct underlying etiology. We excluded gastroschisis cases whose clinical presentations suggested limb-body wall complexes or amniotic band sequences.

We geocoded the addresses reported from residential history during the first month of pregnancy and at birth by using StreetMap Premium North America locators in ArcGIS Pro 3.2.0 (Esri Inc, Redlands, CA). We used the geocode during the first month of pregnancy, though if missing, we used the geocode at birth instead.

Of the initial 4698 participants, we excluded those with missing case/control status (*N* = 2), those with birth defect categories that were not included in both studies (*N* = 1514), those with type 1 or 2 diabetes, or unknown diabetes status (*N* = 77) as an important risk factor of birth defects [18], and those with missing education, multivitamin use, or weekly average 1-h daily maximum temperature (*N* = 100). Our analytic study population included 3005 participants.

### Exposure assessment

Wildfire smoke exposure was estimated using two methods: fire density PM_2.5_ and fire increment PM_2.5_.

#### Primary exposure: fire density PM_2.5_

Wildfire hotspot density was calculated using satellite-detected wildfire hotspots from the Moderate Resolution Imaging Spectroradiometer (MODIS) onboard the Terra satellite [19]. MODIS hotspots were quality controlled to remove low-quality detections as well as detections associated with agricultural land and persistent sources, and have previously been shown to have an R2 of 0.95 when compared to area burned reported by fire agencies [20]. For November 2000 through 2018, wildfire smoke exposure was estimated using a Kernel Density Estimation (KDE) procedure to spatially map daily fire occurrence density with a specified radius of 300 km (after testing sensitivities of 50 km, 100 km, 200 km, 250 km, 300 km, and 500 km in relation to ambient monitoring of PM_2.5_). This nonparametric statistical method for estimating probability densities has been widely used for wildfire occurrence zones [21], and it has the advantage of directly producing density estimates that are not influenced by grid size and localization effects. Hotspot KDE densities were calculated for November 2000 to 2018 using the selected parameters and quality-controlled MODIS hotspots. The resulting raw densities were compared to PM_2.5_ measurements from regulatory ambient air quality monitoring stations obtained from the U.S. Environmental Protection Agency’s Air Quality System. The raw density values were scaled to PM_2.5_ units using a linear regression derived from this comparison. The resulting values were used to generate weekly assignments, and these estimates are referred to below as “fire density PM_2.5_”.

#### Secondary exposure: fire increment PM_2.5_

**Fig. 1:**
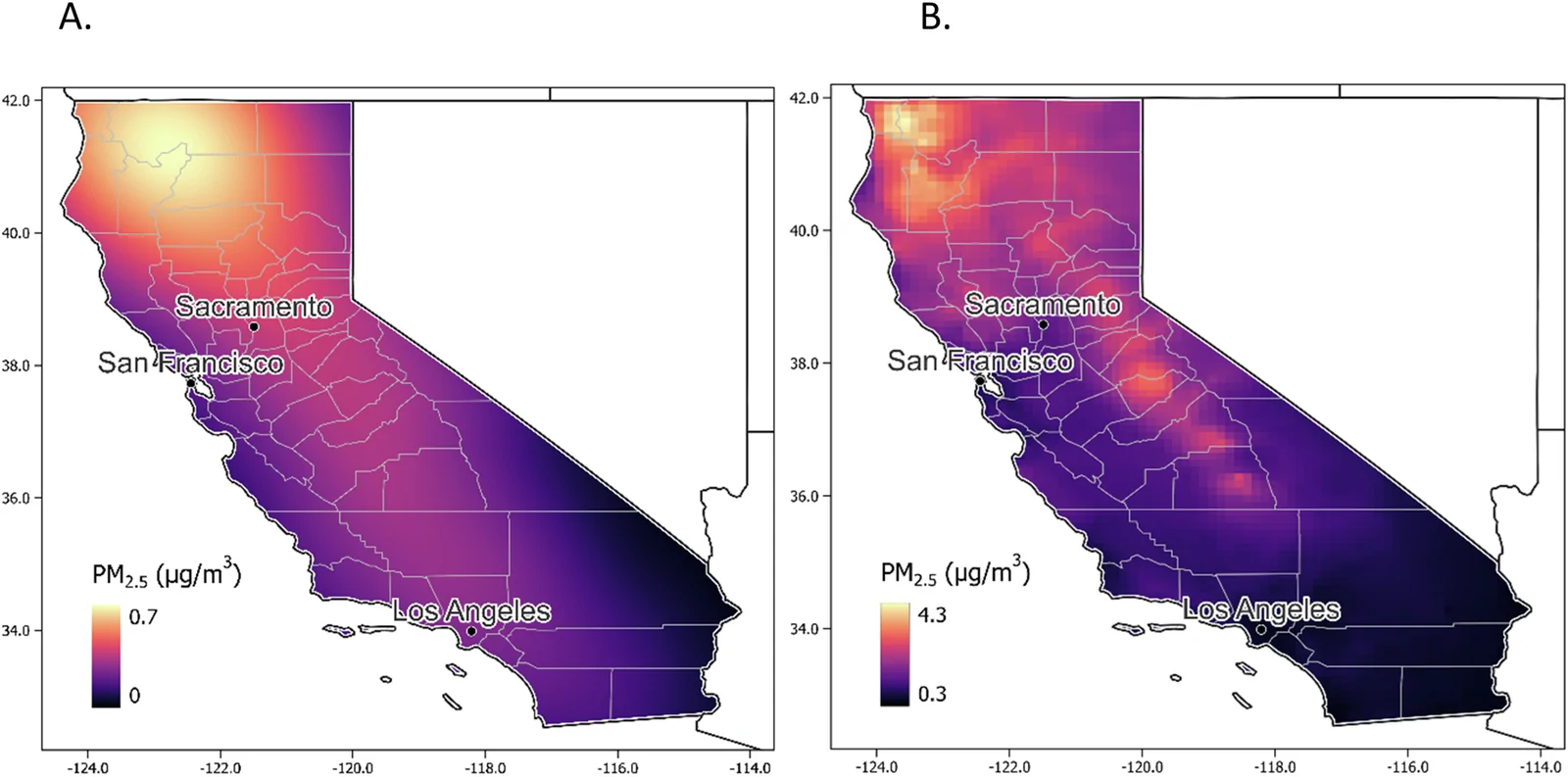
Mean wildfire smoke PM_2.5_ estimates for the full period of data availability in California. **A** fire density PM_2.5_ (2000–2018). **B** fire increment PM_2.5_ (2007–2018).

For a subset of the study population, an additional estimate of wildfire smoke was estimated for the time periods when data were available (2006–2018; *N* = 107; case *n* = 757, control *n* = 314). Ambient PM_2.5_ daily concentrations with and without wildland fire emissions were simulated using the Community Multi-scale Air Quality model (CMAQ) [22]. CMAQ is a three-dimensional Eulerian chemical transport model (CTM) that simulates atmospheric transport and dispersion of VOC, NOx, CO, SO_2_, and PM emissions, as well as the atmospheric chemistry and deposition of gases and aerosols. To improve estimates, raw CMAQ simulation results were bias corrected using daily observations from regulatory air quality monitors obtained from the U.S. Environmental Protection Agency’s Air Quality System. Additional details of the bias correction, including cross-validation results, are published elsewhere [23]. These estimates are referred to below as “fire increment PM_2.5_”. Study period mean fire density PM_2.5_ (2000–2018) and fire increment PM_2.5_ (2007–2018) indicate similar trends of increasing smoke exposure from south to north in California (Fig. 1).

Weekly average of daily fire density PM_2.5_ and fire increment PM_2.5_, along with the highest daily reading for the week, were assigned to each participant in the first 8 weeks of pregnancy from the date of conception to reflect the critical development of selected structural birth defects during this period [24]. To protect participant privacy, assignments were made based on a geocoded location that was shifted between up to 1.1 km in a random direction from the latitude and longitude coordinates of the originally geocoded maternal address. Weekly assignments were aggregated across the first 8 weeks of pregnancy to calculate the mean exposure of fire density PM_2.5_ and fire increment PM_2.5_ based on weekly averages and maximum exposure based on the highest weekly reading. Total PM_2.5_ was estimated using a hybrid ensemble model with 1 km resolution provided by Joel Schwartz at Harvard T.H. Chan School of Public Health [25]. In addition to the fire metrics, we also examined the risk of birth defects in relation to total PM_2.5_ estimates (Harvard) and the non-fire PM_2.5_ from CMAQ for comparison to wildfire metrics. Daily meteorological data were obtained from the GridMet with weekly average 1-hour daily maximum and minimum and mean temperatures gridded surface (4 km) meteorological data provided by Abatzoglou [26].

### Statistical analyses

Mean and maximum fire density PM_2.5_, total PM_2.5_, CMAQ fire increment PM_2.5_, and CMAQ non-fire PM_2.5_ estimates over the first 8 weeks of pregnancy were categorized by quartile. Quartiles were chosen so that the results could be more easily compared with previous studies [4–6], and cut-offs were determined by the distribution in the controls. The odds of each of the 14 birth defects at each quartile were examined in relation to the lowest quartile of exposure for the average and maximum metrics using logistic regression. The control selection process aimed to ensure the control group was representative of the base population; however, we adjusted for the following covariates based on a priori subject matter knowledge [27]: weekly average 1-hour daily maximum temperature, maternal education, folate-containing multivitamin use and smoking in the one month prior to pregnancy or during the first two months of pregnancy. Fire density PM_2.5_, and fire increment, PM_2.5_, exposures were modeled using restricted cubic splines to allow for nonlinear relationships with risk of birth defects. Knots were placed at five distinct intervals: the fifth, 27.5th, 50th, 72.5th, and 95th percentile distribution of the exposure per a priori placement based on previous studies [28].

## Results

The distribution of study population characteristics was examined in relation to case/control status (Table 1). Covariates were generally balanced between cases and controls, including maternal age, education, race/ethnicity, nativity, parity, smoking, vitamin use and infant sex. There were more participants in the first half of the study (2001–2008) than in the second half (2009–2018), but the distribution between cases and controls was balanced. We organize the results by starting with the fire density PM2.5 and the total PM2.5 from the Harvard model in the entire study population, followed by the CMAQ fire- and non-fire-sourced PM2.5 in a subset of the population.

**Table 1:** Characteristics of Cases and Controls from NBDPS and BD-STEPS in California, 2000–2018.

	All		Cases		Controls	
	*N*	%	*N*	%	*N*	%
**Total**	3005		1970		1035	
**Maternal age**						
<20 years	411	13.68	273	13.86	138	13.33
20–24 years	875	29.12	585	29.7	290	28.02
25–29 years	819	27.25	540	27.41	279	26.96
30–24 years	572	19.03	363	18.43	209	20.19
35+ years	328	10.92	209	10.61	119	11.5
**Education**						
0–12 years of education	1805	60.07	1204	61.12	601	58.07
More than 12 years of education	1200	39.93	766	38.88	434	41.93
**Race/ethnicity**						
Non-Hispanic White	757	25.19	485	24.62	272	26.28
Non-Hispanic Black	74	2.46	39	1.98	35	3.38
American Indian or Alaska Native	18	0.6	12	0.61	6	0.58
Hispanic	1868	62.16	1241	62.99	627	60.58
Asian/Other Pacific Islander/Native Hawaiian	148	4.93	101	5.13	47	4.54
Other	139	4.63	92	4.67	47	4.54
Missing	1	0.03			1	0.1
**Maternal birth country**						
Foreign born	1128	37.54	748	37.97	380	36.71
US born	1874	62.36	1219	61.88	655	63.29
Missing	3	0.1	3	0.15		
**Smoking**^a^						
No smoking reported	2612	86.92	1713	86.95	899	86.86
Any smoking reported	393	13.08	257	13.05	136	13.14
**Infant sex**						
Male	1665	55.41	1119	56.8	546	52.75
Female	1334	44.39	845	42.89	489	47.25
Missing	6	0.2	6	0.3		
**Previous livebirths**						
None	1082	36.01	718	36.45	364	35.17
One	892	29.68	581	29.49	311	30.05
Two or more	1030	34.28	671	34.06	359	34.69
Missing	1	0.03			1	0.1
**Season of conception**						
December-February	770	25.62	518	26.29	252	24.35
March-May	735	24.46	474	24.06	261	25.22
June-August	707	23.53	469	23.81	238	23.00
September-November	793	26.39	509	25.84	284	27.44
**Year of birth**						
2001–2003	1032	34.34	627	31.83	405	39.13
2004–2008	1146	38.14	767	38.93	379	36.62
2009–2013	438	14.58	286	14.52	152	14.69
2014–2018	389	12.95	290	14.72	99	9.57
**Any folate vitamin use**^a^						
No	923	30.72	608	30.86	315	30.43
Yes	2082	69.28	1362	69.14	720	69.57

^a^During the 1 month prior to pregnancy and the first 2 months of pregnancy.

In the entire study population, four exposure metrics were examined in relation to birth defect risk by quartile (Table 2). Average and maximum fire density PM_2.5_ was associated with increased risk of cleft lip and coarctation of the aorta (Table 2). For example, the third quartile of average fire density during the first two months of pregnancy was associated with increased odds of cleft lip (OR = 2.29; 95% CI: 1.12, 4.69). Similarly, the highest quartile of maximum fire density PM_2.5_ was associated with increased risk of coarctation of the aorta (OR = 2.26; 95% CI: 1.02, 4.99).

**Table 2:** Adjusted Average/Maximum Fire Density PM_2.5_ and Total PM_2.5_ in relation to odds of structural birth defects.

Exposure	Quartile Range	Spina bifida			Anotia/microtia			Cleft palate			Cleft lip with or without cleft palate		
		Case, *n*	Control, *n*	OR (95% CI)	Case, *n*	Control, *n*	OR (95% CI)	Case, *n*	Control, *n*	OR (95% CI)	Case, *n*	Control, *n*	OR (95% CI)
Average Fire Density PM2.5	0– < 0.04	43	205	1.0 (ref)	29	205	1.0 (ref)	40	205	1.0 (ref)	81	205	1.0 (ref)
	0.04– < 0.08	40	206	0.93 (0.57,1.50)	38	206	1.21 (0.71,2.05)	33	206	0.85 (0.51,1.41)	84	206	1.04 (0.73,1.50)
	0.08– < 0.24	39	206	0.97 (0.57,1.64)	34	206	0.99 (0.55,1.77)	39	206	1.07 (0.63,1.84)	114	206	1.40 (0.96,2.04)
	0.24–5.28	47	206	1.26 (0.69,2.28)	35	206	0.91 (0.47,1.76)	45	206	1.31 (0.71,2.41)	113	206	1.37 (0.89,2.10)
Maximum Fire density PM2.5	0– < 0.52	40	202	1.0 (ref)	30	202	1.0 (ref)	37	202	1.0 (ref)	80	202	1.0 (ref)
	0.52– < 0.95	40	209	0.98 (0.61,1.59)	36	209	1.15 (0.68,1.95)	38	209	1.00 (0.61,1.64)	105	209	1.24 (0.87,1.76)
	0.95– < 2.24	45	206	1.18 (0.73,1.93)	31	206	0.96 (0.55,1.69)	40	206	1.09 (0.66,1.83)	98	206	1.13 (0.78,1.63)
	2.24–62.32	44	206	1.20 (0.68,2.11)	39	206	1.06 (0.58,1.97)	42	206	1.20 (0.67,2.14)	109	206	1.20 (0.79,1.81)
Average Harvard AQ PM2.5	0.76– < 9.41	31	227	1.0 (ref)	38	227	1.0 (ref)	51	227	1.0 (ref)	103	227	1.0 (ref)
	9.41– < 12.54	48	228	1.54 (0.93,2.55)	35	228	0.88 (0.53,1.47)	30	228	**0.59 (0.36,0.97)**	120	228	1.11 (0.80,1.55)
	12.54– < 20.84	51	228	**1.72 (1.06,2.80)**	37	228	1.03 (0.63,1.70)	43	228	0.83 (0.53,1.30)	91	228	0.89 (0.63,1.25)
	20.84–74.71	51	228	**1.79 (1.04,3.06)**	30	228	0.94 (0.52,1.68)	33	228	0.63 (0.37,1.07)	94	228	0.99 (0.68,1.45)
Maximum Harvard AQ PM2.5	2.81– < 18.21	29	227	1.0 (ref)	28	227	1.0 (ref)	45	227	1.0 (ref)	117	227	1.0 (ref)
	18.21– < 29.19	46	228	1.60 (0.97,2.64)	46	228	**1.68 (1.01,2.80)**	34	228	0.76 (0.47,1.23)	95	228	0.80 (0.57,1.11)
	29.19– < 53.72	57	228	**2.27 (1.35,3.82)**	36	228	1.47 (0.84,2.59)	42	228	0.91 (0.56,1.50)	97	228	0.90 (0.64,1.28)
	53.72–199.55	49	228	**2.06 (1.18,3.59)**	30	228	1.27 (0.68,2.37)	36	228	0.78 (0.45,1.34)	99	228	0.98 (0.67,1.44)

Cleft lip^a^			Esophageal atresia			Transverse limb deficiency			Diaphragmatic hernia			Gastroschisis		
Case, *n*	Control, *n*	OR (95% CI)	Case, *n*	Control, *n*	OR (95% CI)	Case, *n*	Control, *n*	OR (95% CI)	Case, *n*	Control, *n*	OR (95% CI)	Case, *n*	Control, *n*	OR (95% CI)
15	172	1.0 (ref)	10	205	1.0 (ref)	13	205	1.0 (ref)	23	205	1.0 (ref)	58	205	1.0 (ref)
28	189	1.83 (0.94,3.58)	17	206	1.65 (0.73,3.73)	15	206	1.20 (0.55,2.62)	26	206	1.19 (0.65,2.18)	55	206	0.91 (0.60,1.39)
32	182	**2.29 (1.12,4.69)**	17	206	1.52 (0.63,3.67)	22	206	2.06 (0.93,4.58)	26	206	1.37 (0.71,2.65)	55	206	0.89 (0.56,1.41)
27	181	1.99 (0.87,4.55)	15	206	1.28 (0.47,3.47)	12	206	1.31 (0.48,3.59)	27	206	1.67 (0.78,3.59)	55	206	0.86 (0.51,1.46)
17	178	1.0 (ref)	13	202	1.0 (ref)	14	202	1.0 (ref)	25	202	1.0 (ref)	59	202	1.0 (ref)
27	182	1.59 (0.83,3.04)	16	209	1.14 (0.53,2.45)	16	209	1.15 (0.55,2.44)	26	209	1.04 (0.58,1.87)	72	209	1.16 (0.77,1.73)
33	183	1.90 (0.99,3.66)	14	206	0.95 (0.42,2.16)	19	206	1.49 (0.70,3.15)	22	206	0.97 (0.52,1.82)	39	206	**0.60 (0.37,0.96)**
25	181	1.44 (0.67,3.09)	16	206	1.00 (0.41,2.43)	13	206	1.11 (0.44,2.79)	29	206	1.49 (0.74,2.97)	53	206	0.76 (0.46,1.25)
32	213	1.0 (ref)	17	227	1.0 (ref)	27	227	1.0 (ref)	20	227	1.0 (ref)	73	227	1.0 (ref)
35	214	1.10 (0.65,1.88)	17	228	0.97 (0.47,1.98)	15	228	0.61 (0.31,1.21)	28	228	1.59 (0.85,2.96)	54	228	0.70 (0.46,1.05)
27	213	0.80 (0.46,1.40)	19	228	1.15 (0.58,2.29)	16	228	0.59 (0.31,1.13)	33	228	1.61 (0.89,2.90)	58	228	0.80 (0.54,1.19)
26	214	0.71 (0.39,1.32)	13	228	0.88 (0.38,2.01)	16	228	0.50 (0.24,1.03)	22	228	0.88 (0.44,1.76)	56	228	0.87 (0.55,1.37)
32	213	1.0 (ref)	17	227	1.0 (ref)	17	227	1.0 (ref)	25	227	1.0 (ref)	67	227	1.0 (ref)
32	214	0.97 (0.57,1.65)	16	228	0.94 (0.46,1.91)	23	228	1.44 (0.75,2.78)	22	228	0.89 (0.49,1.63)	58	228	0.88 (0.59,1.32)
32	213	0.91 (0.52,1.61)	18	228	1.26 (0.60,2.62)	22	228	1.09 (0.53,2.21)	36	228	1.32 (0.74,2.36)	58	228	0.93 (0.60,1.44)
24	214	0.67 (0.35,1.26)	15	228	1.12 (0.49,2.54)	12	228	0.54 (0.23,1.26)	20	228	0.70 (0.35,1.40)	58	228	0.99 (0.62,1.57)

Tetralogy of Fallot			D-Transposition of the great arteries			TAPVR			Hypoplastic left heart syndrome			Coarctation of the aorta		
Case, *n*	Control, *n*	OR (95% CI)	Case, *n*	Control, *n*	OR (95% CI)	Case, *n*	Control, *n*	OR (95% CI)	Case, *n*	Control, *n*	OR (95% CI)	Case, *n*	Control, *n*	OR (95% CI)
24	205	1.0 (ref)	19	205	1.0 (ref)	8	205	1.0 (ref)	26	205	1.0 (ref)	16	205	1.0 (ref)
26	206	1.04 (0.57,1.88)	8	206	**0.41 (0.17,0.97)**	11	206	1.26 (0.49,3.23)	17	206	0.69 (0.36,1.33)	22	206	1.40 (0.71,2.77)
34	206	1.29 (0.69,2.39)	25	206	1.24 (0.61,2.53)	9	206	0.88 (0.31,2.52)	16	206	0.76 (0.37,1.58)	27	206	1.81 (0.89,3.68)
19	206	0.69 (0.32,1.49)	18	206	0.87 (0.37,2.05)	16	206	1.37 (0.46,4.04)	15	206	0.82 (0.35,1.95)	25	206	1.75 (0.78,3.96)
28	202	1.0 (ref)	17	202	1.0 (ref)	12	202	1.0 (ref)	19	202	1.0 (ref)	13	202	1.0 (ref)
25	209	0.85 (0.48,1.52)	17	209	0.92 (0.45,1.87)	7	209	0.54 (0.21,1.40)	26	209	1.40 (0.75,2.63)	31	209	**2.33 (1.18,4.59)**
28	206	0.93 (0.52,1.68)	18	206	0.92 (0.44,1.90)	9	206	0.62 (0.24,1.57)	15	206	0.90 (0.43,1.88)	18	206	1.41 (0.66,3.02)
22	206	0.67 (0.33,1.34)	18	206	0.84 (0.37,1.90)	16	206	0.91 (0.36,2.33)	14	206	1.01 (0.43,2.37)	28	206	**2.26 (1.02,4.99)**
28	227	1.0 (ref)	20	227	1.0 (ref)	15	227	1.0 (ref)	20	227	1.0 (ref)	20	227	1.0 (ref)
28	228	1.06 (0.60,1.88)	21	228	1.00 (0.52,1.94)	8	228	0.49 (0.20,1.21)	18	228	1.08 (0.54,2.13)	28	228	1.37 (0.74,2.55)
36	228	1.28 (0.75,2.18)	17	228	0.86 (0.44,1.68)	16	228	1.12 (0.54,2.33)	20	228	0.91 (0.47,1.75)	25	228	1.28 (0.69,2.38)
23	228	0.76 (0.40,1.44)	17	228	0.92 (0.43,1.96)	7	228	0.59 (0.21,1.64)	17	228	0.57 (0.27,1.19)	22	228	1.22 (0.60,2.47)
30	227	1.0 (ref)	18	227	1.0 (ref)	14	227	1.0 (ref)	15	227	1.0 (ref)	20	227	1.0 (ref)
28	228	0.95 (0.55,1.64)	22	228	1.19 (0.62,2.29)	7	228	0.50 (0.20,1.26)	17	228	1.18 (0.58,2.44)	25	228	1.23 (0.66,2.28)
30	228	0.99 (0.56,1.76)	19	228	1.11 (0.55,2.28)	13	228	1.25 (0.55,2.84)	26	228	1.39 (0.69,2.84)	25	228	1.45 (0.76,2.79)
27	228	0.88 (0.47,1.64)	16	228	0.96 (0.43,2.11)	12	228	1.33 (0.54,3.30)	17	228	0.84 (0.38,1.88)	25	228	1.57 (0.78,3.18)

95% confidence intervals exclude the null value.

Adjusted for weekly average 1-h daily maximum temperature, maternal education, folate-containing multivitamin use and smoking in the one month prior to pregnancy or during the first 2 months of pregnancy.

^a^NBDPS data only.

Average Fire Density PM2.5: Q1: 0– < 0.03875; Q2: 0.03875– < 0.08125; Q3: 0.08125– < 0.236875; Q4: 0.236875–3.26625.

Maximum Fire density PM2.5: Q1: 0.01– < 0.52; Q2: 0.52– < 0.93; Q3: 0.93– < 2.105; Q4: 2.105–62.32.

Average Harvard AQ PM2.5: Q1: 0.75875– < 9.475; Q2: 9.475– < 12.8225; Q3: 12.8225– < 21.03875; Q4: 1.03875–74.70625.

Maximum Harvard AQ PM2.5: Q1: 2.81– < 18.21; Q2: 18.21– < 30.325; Q3: 30.325– < 54.3; Q4: 54.3–199.55.

Additional estimates were suggestive of increased risk for spina bifida, cleft palate, esophageal atresia, transverse limb deficiency, and diaphragmatic hernia, comparing each of the highest three quartiles to the lowest quartile. Associations between average and maximum total PM_2.5_ were consistently associated with spina bifida (average 4th quartile OR = 1.79; 95% CI: 1.04, 3.06 and maximum 4th quartile OR = 2.06; 95% CI: 1.18, 3.59). Higher odds ratios (ranging from 1.2 to 1.5) for coarctation of the aorta were also observed comparing the highest three quartiles to the lowest quartile of average and maximum total PM_2.5_, though estimates were not statistically significant. For hypoplastic left heart syndrome, maximum total PM_2.5_ showed a pattern of elevated odds, but not statistically significant, for the highest three quartiles of exposure.

In a subset of the population where fire and non-fire related PM_2.5_ was available from CMAQ, associations between the average and maximum fire increment were associated with increased odds of spina bifida for all quartiles compared to the lowest (average 3rd quartile OR = 2.28; 95% CI: 1.10, 4.73 and maximum 4th quartile OR = 2.76; 95% CI: 1.14, 6.66) (Table 3). Attenuated but still positive associations were found between each of the highest three quartiles of average and maximum non-fire PM_2.5_ and odds of spina bifida, but estimates were not statistically significant (Table 3).

**Table 3:** Adjusted Average/Maximum Fire Increment PM_2.5_ and CMAQ Non-Fire PM_2.5_ in relation to odds of structural birth defects.

Model	Quartile Range	Spina bifida			Anotia/microtia			Cleft palate			Cleft lip with or without cleft palate		
		Case, *n*	Control, *n*	OR (95% CI)	Case, *n*	Control, *n*	OR (95% CI)	Case, *n*	Control, *n*	OR (95% CI)	Case, *n*	Control, *n*	OR (95% CI)
Average Fire Increment CMAQ	0.005– < 0.23	14	78	1.0 (ref)	14	78	1.0 (ref)	20	78	1.0 (ref)	45	78	1.0 (ref)
	0.23– < 0.60	15	79	1.16 (0.52,2.59)	16	79	1.03 (0.46,2.29)	16	79	0.79 (0.38,1.64)	39	79	0.82 (0.48,1.40)
	0.6– < 1.70	29	78	**2.28 (1.10,4.73)**	18	78	1.15 (0.52,2.53)	24	78	1.19 (0.60,2.35)	52	78	1.11 (0.66,1.85)
	1.70–12.49	24	79	1.84 (0.87,3.91)	16	79	1.01 (0.45,2.28)	17	79	0.83 (0.40,1.72)	44	79	0.91 (0.53,1.54)
Maximum Fire Increment CMAQ	0.02– < 0.83	8	78	1.0 (ref)	13	78	1.0 (ref)	14	78	1.0 (ref)	35	78	1.0 (ref)
	0.83– < 4.17	30	79	**4.17 (1.76,9.84)**	24	79	1.65 (0.77,3.57)	24	79	1.73 (0.82,3.66)	53	79	1.43 (0.83,2.46)
	4.17– < 9.75	23	78	**3.14 (1.31,7.54)**	12	78	0.86 (0.36,2.02)	21	78	1.51 (0.71,3.20)	47	78	1.31 (0.76,2.26)
	9.75–75.06	21	79	**2.76 (1.14,6.66)**	15	79	1.15 (0.51,2.61)	18	79	1.25 (0.58,2.71)	45	79	1.24 (0.72,2.13)
Average Non-Fire CMAQ	3.51– < 6.24	17	78	1.0 (ref)	19	78	1.0 (ref)	17	78	1.0 (ref)	53	78	1.0 (ref)
	6.24– < 7.74	19	79	1.11 (0.54,2.32)	17	79	0.86 (0.41,1.78)	26	79	1.54 (0.77,3.07)	57	79	1.06 (0.65,1.72)
	7.74– < 12.16	20	78	1.24 (0.57,2.73)	18	78	1.14 (0.52,2.48)	19	78	0.98 (0.44,2.18)	39	78	0.67 (0.38,1.19)
	12.16–23.86	26	79	1.94 (0.79,4.76)	10	79	0.82 (0.30,2.30)	15	79	0.68 (0.27,1.70)	31	79	0.47 (0.24,0.91)
Maximum Non-Fire CMAQ	6.28– < 11.59	18	78	1.0 (ref)	20	78	1.0 (ref)	17	78	1.0 (ref)	55	78	1.0 (ref)
	11.59– < 17.93	20	79	1.18 (0.57,2.42)	16	79	0.86 (0.41,1.80)	24	79	1.37 (0.68,2.76)	49	79	0.86 (0.52,1.42)
	17.93– < 27.29	18	78	1.05 (0.46,2.36)	13	78	0.92 (0.40,2.11)	21	78	1.14 (0.51,2.52)	37	78	0.65 (0.36,1.17)
	27.29–50.23	26	79	1.69 (0.68,4.17)	15	79	1.47 (0.56,3.89)	15	79	0.75 (0.30,1.92)	39	79	0.65 (0.33,1.27)

Diaphragmatic hernia			Gastroschisis			Tetralogy of Fallot		
Case, *n*	Control, *n*	OR (95% CI)	Case, *n*	Control, *n*	OR (95% CI)	Case, *n*	Control, *n*	OR (95% CI)
16	78	1.0 (ref)	27	78	1.0 (ref)	15	78	1.0 (ref)
6	79	0.38 (0.14,1.03)	30	79	1.06 (0.57,1.97)	8	79	0.51 (0.20,1.29)
6	78	0.38 (0.14,1.04)	28	78	1.04 (0.55,1.94)	10	78	0.64 (0.26,1.53)
22	79	1.38 (0.65,2.90)	19	79	0.72 (0.36,1.42)	12	79	0.73 (0.31,1.73)
8	78	1.0 (ref)	28	78	1.0 (ref)	14	78	1.0 (ref)
11	79	1.39 (0.52,3.67)	31	79	1.09 (0.59,2.03)	17	79	1.18 (0.53,2.63)
13	78	1.68 (0.65,4.30)	28	78	0.98 (0.53,1.81)	6	78	0.42 (0.15,1.17)
18	79	2.18 (0.89,5.34)	17	79	0.62 (0.31,1.23)	8	79	0.56 (0.22,1.41)
16	78	1.0 (ref)	31	78	1.0 (ref)	21	78	1.0 (ref)
10	79	0.63 (0.27,1.48)	20	79	0.63 (0.33,1.21)	5	79	**0.24 (0.09,0.66)**
13	78	0.74 (0.30,1.82)	27	78	0.84 (0.43,1.62)	14	78	0.59 (0.25,1.37)
11	79	0.50 (0.18,1.43)	26	79	0.83 (0.38,1.79)	5	79	**0.19 (0.06,0.64)**
11	78	1.0 (ref)	23	78	1.0 (ref)	16	78	1.0 (ref)
12	79	1.05 (0.44,2.55)	28	79	1.20 (0.63,2.28)	9	79	0.55 (0.23,1.34)
19	78	1.53 (0.62,3.80)	22	78	1.03 (0.49,2.16)	13	78	0.75 (0.30,1.86)
8	79	0.58 (0.18,1.85)	31	79	1.49 (0.66,3.37)	7	79	0.38 (0.12,1.22)

95% confidence intervals exclude the null value.

Adjusted for weekly average 1-h daily maximum temperature, maternal education, folate-containing multivitamin use and smoking in the one month prior to pregnancy or during the first two months of pregnancy.

We observed additional notable but not statistically significant patterns of elevated odds ratios across all three quartiles of higher exposure, conveying increased risk of several defects. For example, higher odds of anotia/microtia were observed among those in the three highest quartiles of average fire increment PM_2.5_ (Table 3). Similarly, the highest three quartiles of maximum fire increment PM_2.5_ were associated with a higher risk of cleft palate, cleft lip with or without cleft palate, and diaphragmatic hernia (Table 3). Maximum non-fire PM2.5 was associated with higher odds of spina bifida, compared to those in the three highest quartiles compared to the lowest, while for diaphragmatic hernia, the second and third quartiles showed elevated odds.

Unadjusted results were similar to those from adjusted models of fire density PM_2.5_ and total PM_2.5_ (Supplementary Table 1) and CMAQ fire increment PM_2.5_ and non-fire PM_2.5_ (Supplementary Table 2). Results from Tables 2 and 3 are also plotted to show the comparison of results from the fire density PM_2.5_ and the fire increment PM_2.5_ (Supplementary Fig. 1). The results from models using cubic splines also support the spina bifida and coarctation of the aorta associations observed in the quartile assessment of exposures (Supplementary Fig. 2).

## Discussion

Our study of more than 3000 participants, including almost 2000 cases in California over the past two decades, found wildfire smoke exposure associated with elevated risk for selected birth defects, including spina bifida, cleft lip, and coarctation of the aorta; however, the robustness of the estimates varied, and the exposure response was not consistently monotonic. Although the biological mechanisms linking wildfire smoke to individual birth defects have not been established, previous studies linking maternal smoking and ambient air pollution to these outcomes do bolster our findings.

We observed a pattern where the third quartile of exposure often had a higher risk of birth defects compared to the fourth quartile. This finding may be due to misclassification of the exposure and/or behavior changes, such as staying indoors, using air purifiers, or leaving the area, that are more likely to occur at high levels, which may protect pregnant people from exposure.

Our findings of increased risk of spina bifida were among the most consistent across analyses and were consistent with our previous study of traffic-related air pollution and spina bifida risk [4] and recent study of wildfire smoke and spina bifida in California that used statewide records of ICD-9 codes for spina bifida and proximity (15 miles) to wildfires during the first trimester of pregnancy [29].

Our suggested finding of an association between wildfire smoke and cleft lip, cleft lip with or without cleft palate and cleft palate were coherent with previous findings in a large study in Brazil during the same study period; however, the exposure assessment was done by region and trimester, and the categorization of birth defects was larger and more inclusive of distinct defects. Furthermore, cases were ascertained based on birth records and ICD-10 codes [30]. We were unable to confirm previous findings of associations between wildfire smoke and gastroschisis [31], and we did not have sufficient power to investigate atrial septal defects [32].

When comparing wildfire and non-wildfire sources, the findings for spina bifida were the most consistent across both sources of PM_2.5,_ as well as the previous literature. Findings of coarctation of the aorta were consistent across both sources of PM_2.5_, though the increased risk of orofacial clefts was not observed for non-wildfire sources of PM_2.5_. This gradient of consistency across these outcomes contribute is reflected in the confidence of our results. Although the fire density PM_2.5_ was available for the full study period, the fire increment PM_2.5_ would have been preferred as an exposure assessment tool, owing to its incorporation of data from the CMAQ chemical transport model; however, it was reassuring that results were largely similar.

Our study included several potential limitations. The wildfire smoke exposure assignments contain measurement error. Estimation of the emissions and ambient concentrations from wildland biomass burning is significantly more challenging than that from well-controlled anthropogenic emission sources like vehicles and industrial processes. Even though the CMAQ is a comprehensive state-of-the-science chemical transport model, it is applied here with wildfire emission inputs that carry significant uncertainty with respect to quantity, timing, and location. Likewise, wildfire density is a proximity measure that assumes a unidirectional influence around the satellite fire detection point and, therefore, lacks consideration of the influence of meteorological transport on the spatial pattern of exposure. Furthermore, regarding the mother’s activity space, it is unknown how much time each mother spent at her home during the first 2 months of pregnancy. This could lead to potential exposure misclassification if, for example, a mother worked at a location that had different exposure levels or evacuated her residence for days or weeks to avoid high wildfire smoke exposure. Data obtained from retrospective studies can be subject to recall error. It is unknown whether women who did, versus did not, participate in the study were systematically different with respect to air pollution exposure. Last, compared to previous studies using birth records, our study had a smaller sample size.

Strengths of the present study include a rigorous, population-based design and comprehensive case ascertainment. Our study population included terminations and stillbirths not captured in studies of birth records. We have high-quality residential history for early pregnancy compared to most previous studies that rely on the address at birth. Our study benefited from daily wildfire smoke metrics estimated at individual locations. The study also allowed for detailed information to be gathered as potential covariates, specifically during the critical period of the first 8 weeks of pregnancy, including multivitamin use and smoking. These study characteristics limited potential selection bias and confounding.

The biological mechanisms linking wildfire smoke exposure to structural birth defects remain uncertain, but several pathways warrant investigation. Wildfire-specific PM_2.5_ may induce oxidative stress, systemic inflammation, and disruption of placental function, which could impair critical developmental processes during organogenesis. For spina bifida, maternal exposure to PM_2.5_ has been associated with altered folate metabolism, a known risk factor for neural tube defects. Similarly, the observed associations with cleft lip and coarctation of the aorta may be mediated through mechanisms involving hypoxia, vascular abnormalities, or epigenetic modifications triggered by air pollution exposure. Future experimental and molecular studies should focus on identifying specific toxicological effects of wildfire-specific PM_2.5_ components, such as polycyclic aromatic hydrocarbons or heavy metals, on embryonic development.

Our results suggest that exposure to wildfire smoke during the first 8 weeks of pregnancy may contribute to the occurrence of spina bifida and potentially cleft lip and coarctation of the aorta in the San Joaquin Valley of California, which is a highly exposed region of the country. Our results contribute to the body of evidence regarding wildfire exposure and risk of birth defects.

## Supplementary information

## Data Availability

The datasets used for these analyses are not publicly available due to the sensitive nature of these outcomes and protections made to study participants. Aggregated or de-identified data may be available from the corresponding author upon reasonable request and subject to appropriate data use agreements.
