# Supplementary material for: Wildland fire smoke and birth defects in California
Source: J Expo Sci Environ Epidemiol. 2026 Apr 15;36(5):964–73. doi: 10.1038/s41370-026-00885-4 (PMC13466691; doi:10.1038/s41370-026-00885-4)
Supplement: Supplementary file 1 [file 41370_2026_885_MOESM1_ESM.docx]

Supplemental Table 1. Unadjusted Average/Maximum Fire Density PM_2.5_ and Total PM_2.5_ in relation to risk of structural birth defects

| **Exposure** | **Quartile Range** | **Spina bifida** | | | **Anotia/microtia** | | | **Cleft palate** | | | **Cleft lip with or without cleft palate** | | |
| --- | --- | --- | --- | --- | --- | --- | --- | --- | --- | --- | --- | --- | --- |
|  |  | *Case, n* | *Control, n* | *OR (95% CI)* | *Case, n* | *Control, n* | *OR (95% CI)* | *Case, n* | *Control, n* | *OR (95% CI)* | *Case, n* | *Control, n* | *OR (95% CI)* |
| Average Fire Density PM2.5 | 0-<0.04125 | 43 | 205 | 1.0 (ref) | 29 | 205 | 1.0 (ref) | 40 | 205 | 1.0 (ref) | 81 | 205 | 1.0 (ref) |
|  | 0.04125-<0.08375 | 40 | 206 | 0.93 (0.58,1.48) | 38 | 206 | 1.30 (0.77,2.19) | 33 | 206 | 0.82 (0.50,1.35) | 84 | 206 | 1.03 (0.72,1.48) |
|  | 0.08375-<0.24 | 39 | 206 | 0.90 (0.56,1.45) | 34 | 206 | 1.17 (0.69,1.99) | 39 | 206 | 0.97 (0.60,1.57) | 114 | 206 | 1.40 (0.99,1.98) |
|  | 0.24-5.28125 | 47 | 206 | 1.09 (0.69,1.72) | 35 | 206 | 1.20 (0.71,2.04) | 45 | 206 | 1.12 (0.70,1.79) | 113 | 206 | 1.39 (0.98,1.96) |
| Maximum Fire density PM2.5 | 0-<0.52 | 40 | 202 | 1.0 (ref) | 30 | 202 | 1.0 (ref) | 37 | 202 | 1.0 (ref) | 80 | 202 | 1.0 (ref) |
|  | 0.52-<0.95 | 40 | 209 | 0.97 (0.60,1.56) | 36 | 209 | 1.16 (0.69,1.95) | 38 | 209 | 0.99 (0.61,1.62) | 105 | 209 | 1.27 (0.89,1.80) |
|  | 0.95-<2.24 | 45 | 206 | 1.10 (0.69,1.76) | 31 | 206 | 1.01 (0.59,1.74) | 40 | 206 | 1.06 (0.65,1.73) | 98 | 206 | 1.20 (0.84,1.71) |
|  | 2.24-62.32 | 44 | 206 | 1.08 (0.67,1.73) | 39 | 206 | 1.27 (0.76,2.13) | 42 | 206 | 1.11 (0.69,1.80) | 109 | 206 | 1.34 (0.94,1.89) |
| Average Harvard AQ PM2.5 | 0.75875-<9.405 | 31 | 227 | 1.0 (ref) | 38 | 227 | 1.0 (ref) | 51 | 227 | 1.0 (ref) | 103 | 227 | 1.0 (ref) |
|  | 9.405-<12.5425 | 48 | 228 | 1.54 (0.95,2.51) | 35 | 228 | 0.92 (0.56,1.50) | 30 | 228 | **0.59 (0.36,0.95)** | 120 | 228 | 1.16 (0.84,1.60) |
|  | 12.5425-<20.8375 | 51 | 228 | **1.64 (1.01,2.65)** | 37 | 228 | 0.97 (0.59,1.58) | 43 | 228 | 0.84 (0.54,1.31) | 91 | 228 | 0.88 (0.63,1.23) |
|  | 20.8375-74.70625 | 51 | 228 | **1.64 (1.01,2.65)** | 30 | 228 | 0.79 (0.47,1.31) | 33 | 228 | 0.64 (0.40,1.04) | 94 | 228 | 0.91 (0.65,1.27) |
| Maximum Harvard AQ PM2.5 | 2.81-<18.21 | 29 | 227 | 1.0 (ref) | 28 | 227 | 1.0 (ref) | 45 | 227 | 1.0 (ref) | 117 | 227 | 1.0 (ref) |
|  | 18.21-<29.19 | 46 | 228 | 1.58 (0.96,2.60) | 46 | 228 | 1.64 (0.99,2.71) | 34 | 228 | 0.75 (0.46,1.22) | 95 | 228 | 0.81 (0.58,1.12) |
|  | 29.19-<53.72 | 57 | 228 | **1.96 (1.21,3.17)** | 36 | 228 | 1.28 (0.76,2.17) | 42 | 228 | 0.93 (0.59,1.47) | 97 | 228 | 0.83 (0.60,1.14) |
|  | 53.72-199.55 | 49 | 228 | **1.68 (1.03,2.76)** | 30 | 228 | 1.07 (0.62,1.84) | 36 | 228 | 0.80 (0.50,1.28) | 99 | 228 | 0.84 (0.61,1.17) |
| **95% confidence intervals exclude null value** | | | |  |  |  |  |  |  |  |  |  |  |

Supplemental Table 1. (continued)

| **Cleft lip*** | | | **Esophageal atresia** | | | **Transverse limb deficiency** | | | **Diaphragmatic hernia** | | | **Gastroschisis** | | |
| --- | --- | --- | --- | --- | --- | --- | --- | --- | --- | --- | --- | --- | --- | --- |
| *Case, n* | *Control, n* | *OR (95% CI)* | *Case, n* | *Control, n* | *OR (95% CI)* | *Case, n* | *Control, n* | *OR (95% CI)* | *Case, n* | *Control, n* | *OR (95% CI)* | *Case, n* | *Control, n* | *OR (95% CI)* |
| 15 | 172 | 1.0 (ref) | 10 | 205 | 1.0 (ref) | 13 | 205 | 1.0 (ref) | 23 | 205 | 1.0 (ref) | 58 | 205 | 1.0 (ref) |
| 28 | 189 | 1.70 (0.88,3.29) | 17 | 206 | 1.69 (0.76,3.78) | 15 | 206 | 1.15 (0.53,2.47) | 26 | 206 | 1.12 (0.62,2.04) | 55 | 206 | 0.94 (0.62,1.43) |
| 32 | 182 | **2.02 (1.05,3.85)** | 17 | 206 | 1.69 (0.76,3.78) | 22 | 206 | 1.68 (0.83,3.43) | 26 | 206 | 1.12 (0.62,2.04) | 55 | 206 | 0.94 (0.62,1.43) |
| 27 | 181 | 1.71 (0.88,3.33) | 15 | 206 | 1.49 (0.66,3.40) | 12 | 206 | 0.92 (0.41,2.06) | 27 | 206 | 1.17 (0.65,2.10) | 55 | 206 | 0.94 (0.62,1.43) |
| 17 | 178 | 1.0 (ref) | 13 | 202 | 1.0 (ref) | 14 | 202 | 1.0 (ref) | 25 | 202 | 1.0 (ref) | 59 | 202 | 1.0 (ref) |
| 27 | 182 | 1.55 (0.82,2.95) | 16 | 209 | 1.19 (0.56,2.54) | 16 | 209 | 1.10 (0.53,2.32) | 26 | 209 | 1.01 (0.56,1.80) | 72 | 209 | 1.18 (0.79,1.75) |
| 33 | 183 | **1.89 (1.02,3.51)** | 14 | 206 | 1.06 (0.48,2.30) | 19 | 206 | 1.33 (0.65,2.73) | 22 | 206 | 0.86 (0.47,1.58) | 39 | 206 | 0.65 (0.41,1.02) |
| 25 | 181 | 1.45 (0.76,2.77) | 16 | 206 | 1.21 (0.57,2.57) | 13 | 206 | 0.91 (0.42,1.99) | 29 | 206 | 1.14 (0.64,2.01) | 53 | 206 | 0.88 (0.58,1.34) |
| 32 | 213 | 1.0 (ref) | 17 | 227 | 1.0 (ref) | 27 | 227 | 1.0 (ref) | 20 | 227 | 1.0 (ref) | 73 | 227 | 1.0 (ref) |
| 35 | 214 | 1.09 (0.65,1.82) | 17 | 228 | 1.00 (0.50,2.00) | 15 | 228 | 0.55 (0.29,1.07) | 28 | 228 | 1.39 (0.76,2.55) | 54 | 228 | 0.74 (0.50,1.10) |
| 27 | 213 | 0.84 (0.49,1.46) | 19 | 228 | 1.11 (0.56,2.20) | 16 | 228 | 0.59 (0.31,1.12) | 33 | 228 | 1.64 (0.92,2.95) | 58 | 228 | 0.79 (0.54,1.17) |
| 26 | 214 | 0.81 (0.47,1.40) | 13 | 228 | 0.76 (0.36,1.60) | 16 | 228 | 0.59 (0.31,1.12) | 22 | 228 | 1.10 (0.58,2.06) | 56 | 228 | 0.76 (0.52,1.13) |
| 32 | 213 | 1.0 (ref) | 17 | 227 | 1.0 (ref) | 17 | 227 | 1.0 (ref) | 25 | 227 | 1.0 (ref) | 67 | 227 | 1.0 (ref) |
| 32 | 214 | 1.00 (0.59,1.68) | 16 | 228 | 0.94 (0.46,1.90) | 23 | 228 | 1.35 (0.70,2.59) | 22 | 228 | 0.88 (0.48,1.60) | 58 | 228 | 0.86 (0.58,1.28) |
| 32 | 213 | 1.00 (0.59,1.69) | 18 | 228 | 1.05 (0.53,2.10) | 22 | 228 | 1.29 (0.67,2.49) | 36 | 228 | 1.43 (0.83,2.47) | 58 | 228 | 0.86 (0.58,1.28) |
| 24 | 214 | 0.75 (0.43,1.31) | 15 | 228 | 0.88 (0.43,1.80) | 12 | 228 | 0.70 (0.33,1.50) | 20 | 228 | 0.80 (0.43,1.47) | 58 | 228 | 0.86 (0.58,1.28) |
| **95% confidence intervals exclude null value** | | | |  |  |  |  |  |  |  |  |  |  |  |
| *NBDPS data only  Average Fire Density PM2.5: Q1: 0-<0.03875; Q2: 0.03875-<0.08125; Q3: 0.08125-<0.236875; Q4: 0.236875-3.26625  Maximum Fire density PM2.5: Q1: 0.01-<0.52; Q2: 0.52-<0.93; Q3: 0.93-<2.105; Q4: 2.105-62.32  Average Harvard AQ PM2.5: Q1: 0.75875-<9.475; Q2: 9.475-<12.8225; Q3: 12.8225-<21.03875; Q4: 1.03875-74.70625  Maximum Harvard AQ PM2.5: Q1: 2.81-<18.21; Q2: 18.21-<30.325; Q3: 30.325-<54.3; Q4: 54.3-199.55 | | | | | | | | | | | | | | |

Supplemental Table 1. (continued)

| **Tetralogy of Fallot** | | | **D-Transposition of the great arteries** | | | **TAPVR** | | | **Hypoplastic left heart syndrome** | | | **Coarctation of the aorta** | | |
| --- | --- | --- | --- | --- | --- | --- | --- | --- | --- | --- | --- | --- | --- | --- |
| *Case, n* | *Control, n* | *OR (95% CI)* | *Case, n* | *Control, n* | *OR (95% CI)* | *Case, n* | *Control, n* | *OR (95% CI)* | *Case, n* | *Control, n* | *OR (95% CI)* | *Case, n* | *Control, n* | *OR (95% CI)* |
| 24 | 205 | 1.0 (ref) | 19 | 205 | 1.0 (ref) | 8 | 205 | 1.0 (ref) | 26 | 205 | 1.0 (ref) | 16 | 205 | 1.0 (ref) |
| 26 | 206 | 1.08 (0.60,1.94) | 8 | 206 | **0.42 (0.18,0.98)** | 11 | 206 | 1.37 (0.54,3.47) | 17 | 206 | 0.65 (0.34,1.24) | 22 | 206 | 1.37 (0.70,2.68) |
| 34 | 206 | 1.41 (0.81,2.46) | 25 | 206 | 1.31 (0.70,2.45) | 9 | 206 | 1.12 (0.42,2.96) | 16 | 206 | 0.61 (0.32,1.18) | 27 | 206 | 1.68 (0.88,3.21) |
| 19 | 206 | 0.79 (0.42,1.48) | 18 | 206 | 0.94 (0.48,1.85) | 16 | 206 | 1.99 (0.83,4.75) | 15 | 206 | 0.57 (0.30,1.12) | 25 | 206 | 1.55 (0.81,3.00) |
| 28 | 202 | 1.0 (ref) | 17 | 202 | 1.0 (ref) | 12 | 202 | 1.0 (ref) | 19 | 202 | 1.0 (ref) | 13 | 202 | 1.0 (ref) |
| 25 | 209 | 0.86 (0.49,1.53) | 17 | 209 | 0.97 (0.48,1.95) | 7 | 209 | 0.56 (0.22,1.46) | 26 | 209 | 1.32 (0.71,2.46) | 31 | 209 | 2.30 (1.17,4.53) |
| 28 | 206 | 0.98 (0.56,1.71) | 18 | 206 | 1.04 (0.52,2.07) | 9 | 206 | 0.74 (0.30,1.78) | 15 | 206 | 0.77 (0.38,1.57) | 18 | 206 | 1.36 (0.65,2.84) |
| 22 | 206 | 0.77 (0.43,1.39) | 18 | 206 | 1.04 (0.52,2.07) | 16 | 206 | 1.31 (0.60,2.83) | 14 | 206 | 0.72 (0.35,1.48) | 28 | 206 | **2.11 (1.06,4.19)** |
| 28 | 227 | 1.0 (ref) | 20 | 227 | 1.0 (ref) | 15 | 227 | 1.0 (ref) | 20 | 227 | 1.0 (ref) | 20 | 227 | 1.0 (ref) |
| 28 | 228 | 1.00 (0.57,1.73) | 21 | 228 | 1.05 (0.55,1.98) | 8 | 228 | 0.53 (0.22,1.28) | 18 | 228 | 0.90 (0.46,1.74) | 28 | 228 | 1.39 (0.76,2.55) |
| 36 | 228 | 1.28 (0.76,2.17) | 17 | 228 | 0.85 (0.43,1.66) | 16 | 228 | 1.06 (0.51,2.20) | 20 | 228 | 1.00 (0.52,1.90) | 25 | 228 | 1.24 (0.67,2.30) |
| 23 | 228 | 0.82 (0.46,1.46) | 17 | 228 | 0.85 (0.43,1.66) | 7 | 228 | 0.46 (0.19,1.16) | 17 | 228 | 0.85 (0.43,1.66) | 22 | 228 | 1.10 (0.58,2.06) |
| 30 | 227 | 1.0 (ref) | 18 | 227 | 1.0 (ref) | 14 | 227 | 1.0 (ref) | 15 | 227 | 1.0 (ref) | 20 | 227 | 1.0 (ref) |
| 28 | 228 | 0.93 (0.54,1.61) | 22 | 228 | 1.22 (0.64,2.33) | 7 | 228 | 0.50 (0.20,1.26) | 17 | 228 | 1.13 (0.55,2.31) | 25 | 228 | 1.24 (0.67,2.30) |
| 30 | 228 | 1.00 (0.58,1.71) | 19 | 228 | 1.05 (0.54,2.05) | 13 | 228 | 0.92 (0.43,2.01) | 26 | 228 | 1.73 (0.89,3.34) | 25 | 228 | 1.24 (0.67,2.30) |
| 27 | 228 | 0.90 (0.52,1.56) | 16 | 228 | 0.88 (0.44,1.78) | 12 | 228 | 0.85 (0.39,1.89) | 17 | 228 | 1.13 (0.55,2.31) | 25 | 228 | 1.24 (0.67,2.30) |
| **95% confidence intervals exclude null value** | | | |  |  |  |  |  |  |  |  |  |  |  |

Supplemental Table 2. Unadjusted Average/Maximum Fire Increment PM_2.5_ and CMAQ non-fire PM_2.5_ in relation to risk of structural birth defects

| **Model** | **Quartile Range** | **Spina bifida** | | | **Anotia/microtia** | | | **Cleft palate** | | | **Cleft lip with or without cleft palate** | | |
| --- | --- | --- | --- | --- | --- | --- | --- | --- | --- | --- | --- | --- | --- |
|  |  | *Case, n* | *Control, n* | *OR (95% CI)* | *Case, n* | *Control, n* | *OR (95% CI)* | *Case, n* | *Control, n* | *OR (95% CI)* | *Case, n* | *Control, n* | *OR (95% CI)* |
| Average Fire Increment CMAQ | 0.005-<0.22875 | 14 | 78 | 1.0 (ref) | 14 | 78 | 1.0 (ref) | 20 | 78 | 1.0 (ref) | 45 | 78 | 1.0 (ref) |
|  | 0.22875-<0.601875 | 15 | 79 | 1.06 (0.48,2.34) | 16 | 79 | 1.13 (0.52,2.47) | 16 | 79 | 0.79 (0.38,1.64) | 39 | 79 | 0.86 (0.50,1.45) |
|  | 0.601875-<1.6975 | 29 | 78 | **2.07 (1.02,4.22)** | 18 | 78 | 1.29 (0.60,2.76) | 24 | 78 | 1.20 (0.61,2.35) | 52 | 78 | 1.16 (0.70,1.92) |
|  | 1.6975-12.48875 | 24 | 79 | 1.69 (0.82,3.51) | 16 | 79 | 1.13 (0.52,2.47) | 17 | 79 | 0.84 (0.41,1.72) | 44 | 79 | 0.97 (0.57,1.62) |
| Maximum Fire Increment CMAQ | 0.02-<0.83 | 8 | 78 | 1.0 (ref) | 13 | 78 | 1.0 (ref) | 14 | 78 | 1.0 (ref) | 35 | 78 | 1.0 (ref) |
|  | 0.83-<4.17 | 30 | 79 | **3.70 (1.60,8.58)** | 24 | 79 | 1.82 (0.87,3.84) | 24 | 79 | 1.69 (0.82,3.51) | 53 | 79 | 1.50 (0.88,2.54) |
|  | 4.17-<9.75 | 23 | 78 | **2.87 (1.21,6.82)** | 12 | 78 | 0.92 (0.40,2.15) | 21 | 78 | 1.50 (0.71,3.16) | 47 | 78 | 1.34 (0.78,2.30) |
|  | 9.75-75.06 | 21 | 79 | **2.59 (1.08,6.20)** | 15 | 79 | 1.14 (0.51,2.55) | 18 | 79 | 1.27 (0.59,2.73) | 45 | 79 | 1.27 (0.74,2.18) |
| Average Non-Fire CMAQ | 3.51-<6.23625 | 17 | 78 | 1.0 (ref) | 19 | 78 | 1.0 (ref) | 17 | 78 | 1.0 (ref) | 53 | 78 | 1.0 (ref) |
|  | 6.23625-<7.73625 | 19 | 79 | 1.10 (0.53,2.28) | 17 | 79 | 0.88 (0.43,1.82) | 26 | 79 | 1.51 (0.76,3.00) | 57 | 79 | 1.06 (0.65,1.73) |
|  | 7.73625-<12.1575 | 20 | 78 | 1.18 (0.57,2.41) | 18 | 78 | 0.95 (0.46,1.94) | 19 | 78 | 1.12 (0.54,2.31) | 39 | 78 | 0.74 (0.44,1.24) |
|  | 12.1575-23.8625 | 26 | 79 | 1.51 (0.76,3.00) | 10 | 79 | 0.52 (0.23,1.19) | 15 | 79 | 0.87 (0.41,1.87) | 31 | 79 | **0.58 (0.34,0.99)** |
| Maximum Non-Fire CMAQ | 6.28-<11.59 | 18 | 78 | 1.0 (ref) | 20 | 78 | 1.0 (ref) | 17 | 78 | 1.0 (ref) | 55 | 78 | 1.0 (ref) |
|  | 11.59-<17.93 | 20 | 79 | 1.10 (0.54,2.23) | 16 | 79 | 0.79 (0.38,1.64) | 24 | 79 | 1.39 (0.70,2.79) | 49 | 79 | 0.88 (0.54,1.44) |
|  | 17.93-<27.29 | 18 | 78 | 1.00 (0.48,2.06) | 13 | 78 | 0.65 (0.30,1.40) | 21 | 78 | 1.24 (0.61,2.52) | 37 | 78 | 0.67 (0.40,1.13) |
|  | 27.29-50.23 | 26 | 79 | 1.43 (0.72,2.81) | 15 | 79 | 0.74 (0.35,1.55) | 15 | 79 | 0.87 (0.41,1.87) | 39 | 79 | 0.70 (0.42,1.17) |
| **95% confidence intervals exclude null value** | | | | |  |  |  |  |  |  |  |  |  |

Supplemental Table 2. (continued)

| **Diaphragmatic hernia** | | | **Gastroschisis** | | | **Tetralogy of Fallot** | | |
| --- | --- | --- | --- | --- | --- | --- | --- | --- |
| *Case, n* | *Control, n* | *OR (95% CI)* | *Case, n* | *Control, n* | *OR (95% CI)* | *Case, n* | *Control, n* | *OR (95% CI)* |
| 16 | 78 | 1.0 (ref) | 27 | 78 | 1.0 (ref) | 15 | 78 | 1.0 (ref) |
| 6 | 79 | 0.37 (0.14,1.00) | 30 | 79 | 1.10 (0.60,2.01) | 8 | 79 | 0.53 (0.21,1.31) |
| 6 | 78 | 0.38 (0.14,1.01) | 28 | 78 | 1.04 (0.56,1.92) | 10 | 78 | 0.67 (0.28,1.57) |
| 22 | 79 | 1.36 (0.66,2.78) | 19 | 79 | 0.69 (0.36,1.35) | 12 | 79 | 0.79 (0.35,1.80) |
| 8 | 78 | 1.0 (ref) | 28 | 78 | 1.0 (ref) | 14 | 78 | 1.0 (ref) |
| 11 | 79 | 1.36 (0.52,3.56) | 31 | 79 | 1.09 (0.60,1.99) | 17 | 79 | 1.20 (0.55,2.60) |
| 13 | 78 | 1.62 (0.64,4.14) | 28 | 78 | 1.00 (0.54,1.84) | 6 | 78 | 0.43 (0.16,1.17) |
| 18 | 79 | 2.22 (0.91,5.41) | 17 | 79 | 0.60 (0.30,1.18) | 8 | 79 | 0.56 (0.22,1.42) |
| 16 | 78 | 1.0 (ref) | 31 | 78 | 1.0 (ref) | 21 | 78 | 1.0 (ref) |
| 10 | 79 | 0.62 (0.26,1.44) | 20 | 79 | 0.64 (0.33,1.21) | 5 | 79 | **0.24 (0.08,0.65)** |
| 13 | 78 | 0.81 (0.37,1.80) | 27 | 78 | 0.87 (0.48,1.59) | 14 | 78 | 0.67 (0.32,1.41) |
| 11 | 79 | 0.68 (0.30,1.56) | 26 | 79 | 0.83 (0.45,1.52) | 5 | 79 | 0.24 (0.08,0.65) |
| 11 | 78 | 1.0 (ref) | 23 | 78 | 1.0 (ref) | 16 | 78 | 1.0 (ref) |
| 12 | 79 | 1.08 (0.45,2.59) | 28 | 79 | 1.20 (0.64,2.27) | 9 | 79 | 0.56 (0.23,1.33) |
| 19 | 78 | 1.73 (0.77,3.87) | 22 | 78 | 0.96 (0.49,1.86) | 13 | 78 | 0.81 (0.37,1.80) |
| 8 | 79 | 0.72 (0.27,1.88) | 31 | 79 | 1.33 (0.71,2.48) | 7 | 79 | 0.43 (0.17,1.11) |
| **95% confidence intervals exclude null value** | | | |  |  |  |  |  |

Supplemental Figure 1. Plotted estimates of association between fire smoke in relation to odds of birth defects across quartiles of exposure (A. Average fire density PM_2.5_ and fire increment PM_2.5_ and B. Maximum fire density PM_2.5_ and fire increment PM_2.5_) using logistic regression.

A.

B.

Supplemental Figure 2. Plotted spline estimates of association between fire smoke in relation to odds of birth defects with knots placed at five distinct intervals: the fifth, 27.5^th^, 50^th^, 72.5^th^, and 95^th^ percentile distribution. (A. Average fire density PM_2.5_; B. Average fire increment PM_2.5_; C. Maximum fire density PM_2.5_; D. Maximum fire increment PM_2.5_)

A.

B.

C.

D.
